# Supplementary material for: A protocol for a critical realist systematic synthesis of interventions to promote pupils’ wellbeing by improving the school climate in low- and middle-income countries
Source: PLoS One. 2024 May 15;19(5):e0286489. doi: 10.1371/journal.pone.0286489 (PMC11095672; doi:10.1371/journal.pone.0286489)
Supplement: S1 File — (DOCX) [file pone.0286489.s002.docx]

Supporting information 2: Search strategy

Records identified from:

Websites (n = )

Organisations (n = )

Citation searching (n = )

etc.

Records removed *before screening*:

Duplicate records removed (n = )

Records marked as ineligible by automation tools (n = )

Records removed for other reasons (n = )

Records identified from*:

Databases (n = )

Registers (n = )

**Identification**

Records screened

(n = )

Records excluded**

(n = )

Reports not retrieved

(n = )

Reports sought for retrieval

(n = )

Reports sought for retrieval

(n = )

Reports not retrieved

(n = )

**Screening**

Reports assessed for eligibility

(n = )

Reports excluded:

Reason 1 (n = )

Reason 2 (n = )

Reason 3 (n = )

etc.

Reports assessed for eligibility

(n = )

Reports excluded:

Reason 1 (n = )

Reason 2 (n = )

Reason 3 (n = )

etc.

Studies included in review

(n = )

Reports of included studies

(n = )

**Included**

*Source:* Page MJ, McKenzie JE, Bossuyt PM, Boutron I, Hoffmann TC, Mulrow CD, et al. The PRISMA 2020 statement: an updated guideline for reporting systematic reviews. BMJ 2021;372:n71. doi: 10.1136/bmj.n71. For more information, visit: <http://www.prisma-statement.org/>
